# Inhibition of HSF1 and SAFB Granule Formation Enhances Apoptosis Induced by Heat Stress

**DOI:** 10.3390/ijms22094982

**Published:** 2021-05-07

**Authors:** Kazunori Watanabe, Takashi Ohtsuki

**Affiliations:** Graduate School of Interdisciplinary Science and Engineering in Health Systems, Okayama University, Okayama 7008530, Japan; ohtsuk@okayama-u.ac.jp

**Keywords:** heat shock response, nuclear stress bodies, HSF1 granules, SAFB granules, liquid-liquid phase separation

## Abstract

Stress resistance mechanisms include upregulation of heat shock proteins (HSPs) and formation of granules. Stress-induced granules are classified into stress granules and nuclear stress bodies (nSBs). The present study examined the involvement of nSB formation in thermal resistance. We used chemical compounds that inhibit heat shock transcription factor 1 (HSF1) and scaffold attachment factor B (SAFB) granule formation and determined their effect on granule formation and HSP expression in HeLa cells. We found that formation of HSF1 and SAFB granules was inhibited by 2,5-hexanediol. We also found that suppression of HSF1 and SAFB granule formation enhanced heat stress-induced apoptosis. In addition, the upregulation of HSP27 and HSP70 during heat stress recovery was suppressed by 2,5-hexanediol. Our results suggested that the formation of HSF1 and SAFB granules was likely to be involved in the upregulation of HSP27 and HSP70 during heat stress recovery. Thus, the formation of HSF1 and SAFB granules was involved in thermal resistance.

## 1. Introduction

Eukaryotic cells possess stress response mechanisms that are induced by environmental stresses, including heat, radiation, and chemical reagents [1,2]. Stress response mechanisms are classified into cell-death inducing and stress resistance mechanisms. Cells maintain homeostasis by selectively activating these two mechanisms, depending on the strength of environmental stress or the concentration of chemical reagents. The major stress resistance mechanisms include translational arrest, upregulation of heat shock proteins (HSPs), and formation of granules [3,4,5,6].

Transient receptor potential (TRP) channels are a superfamily of cation channels, which undergo a closed-to-open gating transition in response to various physical and chemical stimuli, including heat [7]. Transient receptor potential vanilloid (TRPV) 1, which is a member of the TRP channel family, can sense temperature (>42 °C) [7,8]. TRPV1 is induced to undergo a conformational change with heat stress, and then, the calcium concentration is increased [8,9]. Many researchers have identified TRPV1 antagonists, such as SB366791 [10], and agonists, such as capsaicin [11].

Granules induced by stresses are classified into stress granules (SGs) and P-bodies that are formed in the cytoplasm, and nuclear stress bodies (nSBs) that are formed in the nucleus. Although SGs and nSBs are rarely detectable in unstressed cells, these granules are present during stresses such as heat stress [5,12]. SGs and P-bodies are detected in several eukaryote cells, while nSBs are detected only in primate cells [13,14,15,16].

nSBs are composed of many RNA-binding proteins and non-coding RNAs [5]. The major protein components of nSBs are heat shock transcription factor (HSF)1 [13,17], HSF2 [18], scaffold attachment factor B (SAFB) [19], SRC-associated during mitosis, 68 kDa protein (SAM68) [20], and splicing factor 2/alternative splicing factor (SF2/ASF) [21]. The non-coding RNA components of nSBs are Satellite III long non-coding RNA (Sat III RNA) [22], initiator tRNA, and elongator tRNA [23].

Many researchers have shown that membraneless structures and nuclear bodies, such as stress granules and paraspeckles, are formed via liquid–liquid phase separation (LLPS) [24,25,26,27]. LLPS is mediated by multivalent weak interactions conferred by a low complexity sequence domain of RNA-binding proteins. Aliphatic alcohols, such as 1,6-hexanediol (1,6-HD) and 2,5-hexanediol (2,5-HD), have been reported to be chemical reagents that inhibit the formation of granules. 1,6-HD and 2,5-HD induce the disruption of hydrophobic interactions in protein structures and subsequent dissolution of the granules. For example, HSF1 granule formation induced by MG132 is inhibited by 10% 1,6-HD [28].

Several functions of proteins and RNAs included in nSBs have been identified. HSF1 and SAFB mainly regulate the expression of HSP70 and HSP27, respectively [29,30]. Bromodomain-containing protein 4 (BRD4) and Sat III RNA are involved in intron retention of mRNA under heat stress and during heat stress recovery [31,32]. Furthermore, the combination of HSF1 knockdown and cisplatin enhanced cell viability inhibition by heat stress in HeLa cells [33]. In addition, nSBs are transiently formed due to heat stress and disappear during the recovery period [5]. These reports suggest the involvement of nSBs in thermal resistance. However, they only describe the functions of the protein- and RNA-components of nSBs; there is no direct evidence that the formation of nSBs is involved in thermal resistance. Therefore, the present study investigated the involvement of nSB formation in thermal resistance by determining the effect of inhibitor compounds on granule formation and HSP expression in vitro.

## 2. Results

### 2.1. Thermal Resistance was Reduced by HSF1 and SAFB Knockdown

To determine whether nSB formation was involved in thermal resistance, the nSB components HSF1, HSF2, SAFB, SAM68, and SF2/ASF were knocked down in HeLa cells. The knockdown efficiencies of these protein components were approximately 80% (Figure S1A,B).

**Figure 1:**
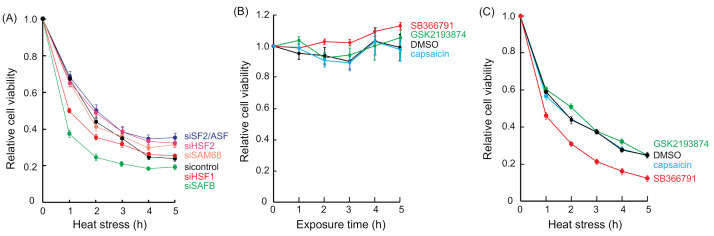
Effect of siRNA and SB366791 treatment on thermal resistance in HeLa cells. (**A**) nSB component knocked-down cells were incubated at 43 °C for 0–5 h. Black, sicontrol; red, siHSF1; pink, siHSF2; green, siSAFB; pale orange, siSAM68; blue, siSF2/ASF. Data represent the means ± SEMs of six independent experiments. The cells were incubated at 37 °C (**B**) or 43 °C (**C**) for 0–5 h in the presence of DMSO (black), capsaicin (light blue), SB366791 (red), or GSK2193874 (green). After treatment, the cells were incubated at 37 °C for 24 h, and then cell viability was analyzed. Data represent the means ± SEMs of six independent experiments.

We performed cell viability assays to determine whether cell growth inhibition induced by heat stress was enhanced by the knockdown of nSB components. As shown in Figure 1A, the cell growth inhibition in non-targeting siRNA (sicontrol)-treated cells was similar to that observed in siHSF2-, siSAM-, and siSF2/ASF-treated cells. By contrast, the cell growth inhibition observed in siHSF1- and siSAFB-treated cells was greater than that observed in sicontrol-treated cells. These findings indicated that HSF1 and SAFB were involved in thermal resistance.

### 2.2. TRPV1 Antagonist, SB366791, Enhanced Cell Growth Inhibition Induced by Heat Stress

TRPV1 can sense temperature [7,8]. In addition, nSBs are formed heat-dependently. Therefore, we speculated that nSB formation would be inhibited by a TRPV1 antagonist. We selected SB366791 for this [10]. The number of HSF1 granules in the presence of SB366791 was similar to that in the presence of DMSO (only the solvent; Figure S2). In contrast, the number of SAFB granules in the presence of SB366791 was lower than that in the presence of DMSO. This result suggested that SB366791 inhibited only SAFB granule formation.

Next, we examined whether heat stress-induced cell growth inhibition was enhanced by SB366791. Capsaicin, a TRPV1 agonist [11], and GSK2193874, a TRPV4 antagonist [34], were used as controls. None of these compounds inhibited cell viability at 37 °C (Figure 1B). In addition, cell growth inhibition induced by heat stress in the presence of capsaicin and GSK2193874 was similar to that observed in the presence of DMSO (Figure 1C). However, the cell growth inhibition induced by heat stress in the presence of SB366791 was greater than that in the presence of DMSO. SB366791, which prevented SAFB granule formation, enhanced heat stress-induced cell growth inhibition, indicating that SAFB granule formation was related to thermal resistance.

### 2.3. 2,5-HD Inhibited HSF1 and SAFB Granule Formation Induced by Heat Stress

HSF1 and SAFB regulate the expression of HSPs involved in thermal resistance under heat stress [29,35]. In addition, TRPV1 is also involved in regulating the expression of HSPs during heat stress; SB366791 decreases the expression levels of HSPs [11,36]. Therefore, siHSF1, siSAFB, and SB366791 treatment might affect the expression of HSPs and other factors involved in thermal resistance. Thus, to determine the involvement of HSF1 and SAFB granule formation in thermal resistance, it was necessary to inhibit the formation of nSBs alone, without affecting the expression of HSPs and other factors involved in thermal resistance.

**Figure 2:**
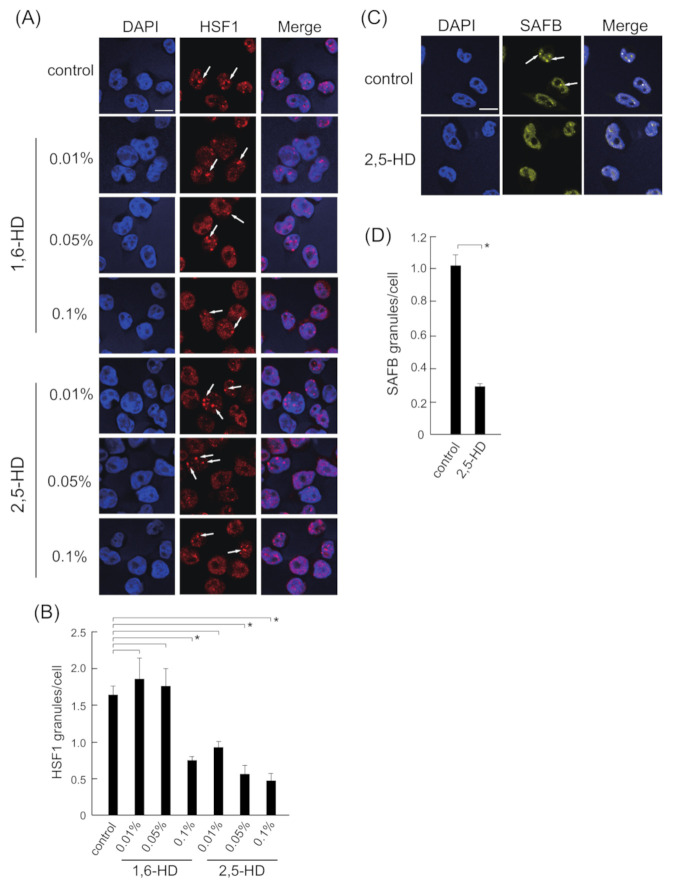
Effect of 2,5-HD on HSF1 and SAFB granule formation. (**A**) HeLa cells were exposed to heat stress at 43 °C for 1 h in the presence of each concentration of 1,6-HD or 2,5-HD. Control cells were cultured in the absence of these compounds. HeLa cells were stained for HSF1 (red) and 4′,6-diamidino-2-phenylindole (DAPI) (blue). The arrows indicate HSF1 granules. Scale bars = 10 µm. (**B**) The number of HSF1 granules per cell is shown. More than 250 cells were counted in each experiment. Data represent the means ± SEMs of four independent experiments. *p*-values were calculated using one-way ANOVA and Dunnett’s test by comparing control with 1,6-HD and 2,5-HD. (**C**) HeLa cells were treated with 0.1% 2,5-HD at 43 °C for 1 h. Control cells were cultured the absence of 2,5-HD. HeLa cells were stained for SAFB (yellow) and DAPI (blue). The arrows indicate SAFB granules. Scale bars = 10 µm. (**D**) The number of SAFB granules per cell is shown. More than 250 cells were counted in each experiment. Data represent the means ± SEM of four independent experiments. * *p* < 0.05; P-values were calculated using one-way ANOVA and Dunnett’s test by comparing control with 2,5-HD.

The formation of membraneless structures and nuclear bodies, such as stress granules and paraspeckles, is inhibited by 1,6-HD and 2,5-HD [24,25,26,27]. Therefore, we examined whether 1,6-HD or 2,5-HD inhibited the heat stress-induced formation of HSF1 granules by immunostaining (Figure 2A,B). The formation of HSF1 granules was inhibited in the presence of 0.1% 1,6-HD as well as 0.01% and 0.05 % 2,5-HD; however, maximum inhibition was detected in the presence of 0.1% 2,5-HD. By contrast, the cells were floated in the presence of 0.25% 2,5-HD under heat stress, indicating cells were damaged (Figure S3). Therefore, 0.1% 2,5-HD was used in subsequent experiments.

Next, we examined whether the formation of SAFB granules was inhibited in the presence of 0.1% 2,5-HD at 43 °C and found it to be inhibited by heat stress (Figure 2C,D). These results suggested that HSF1 and SAFB granules induced by heat stress were probably formed via LLPS.

### 2.4. 2,5-HD Enhanced Cell Growth Inhibition Induced by Heat Stress

**Figure 3:**
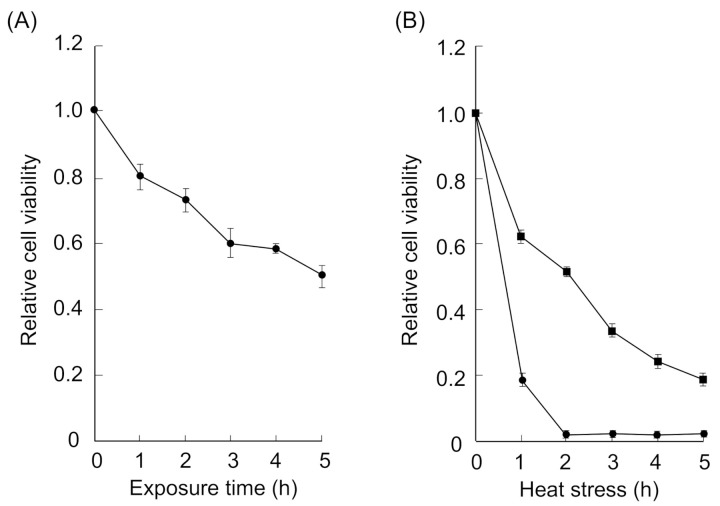
Effects on thermal resistance in the presence of 0.1% 2,5-HD. (**A**) The cells were incubated at 37 °C in the presence of 0.1% 2,5-HD. After treatment, the cells were incubated at 37 °C for 24 h, and then, cell viability was analyzed. Data represent the means ± SEMs of six independent experiments. The cell viability of cells in the absence of 2,5-HD was defined as 1.0. (**B**) The cells were incubated at 43 °C in the presence of 0.1% 2,5-HD (circle) or in the absence of 2,5-HD (square). After treatment, the cells were incubated at 37 °C for 24 h, and then, cell viability was analyzed. Data represent the means ± SEMs of six independent experiments.

To determine whether the formation of HSF1 and SAFB granules was involved in thermal resistance, we measured cell viability in the presence of 2,5-HD. The viability of cells treated with 0.1% 2,5-HD at 37 °C was time-dependently inhibited (Figure 3A), demonstrating the cytotoxicity of this treatment after a long exposure. In addition, the viability of cells treated with 0.1% 2,5-HD at 43 °C was lower than that in the absence of 2,5-HD (Figure 3B).

Although heat stress-induced cell growth inhibition was enhanced in the presence of 0.1% 2,5-HD, these results alone did not indicate that the formation of HSF1 and SAFB granules was important for thermal resistance, as thermal resistance may be affected via other mechanisms. As shown in previous reports, HSF1 activation, which is induced by phosphorylation at Ser326; HSP expression; and SG formation are involved in thermal resistance [4,29,30,35,37,38,39]. However, it is unclear if these three events are affected in the presence of 0.1% 2,5-HD under heat stress. Therefore, it was necessary to eliminate the effect of 2,5-HD on these thermal resistance mechanisms.

We first examined whether the formation of eIF3 γ granules, which localize into SGs, was inhibited by 0.1% 2,5-HD and found it to be unaffected (Figure S4A,B). Next, we examined the effect of 0.1% 2,5-HD on the phosphorylation of HSF1 at Ser326 and the expression of HSF1, HSP70, and HSP27 (Figure S4C,D). The expression levels of HSP27 and HSP70 under heat stress in the presence of 0.1% 2,5-HD were similar to those in its absence. Moreover, the phosphorylation level of HSF1 at Ser326 under heat stress in the presence of 0.1% 2,5-HD was also similar to that in its absence. However, the expression level of HSF1 under heat stress in the absence of 2,5-HD was slightly higher than that in the presence of 0.1% 2,5-HD. These results indicated that heat-induced phenomena, except HSF1 and SAFB granule formation, were either not or only slightly affected by treatment with 0.1% 2,5-HD. In summary, 2,5-HD affects the formation of HSF1 and SAFB granules without affecting the expression of HSP27 and HSP70, phosphorylation of HSF1, or formation of SGs.

### 2.5. Inhibition of HSF1 and SAFB Granule Formation Enhanced Temperature-Dependent Cell Growth Inhibition

**Figure 4:**
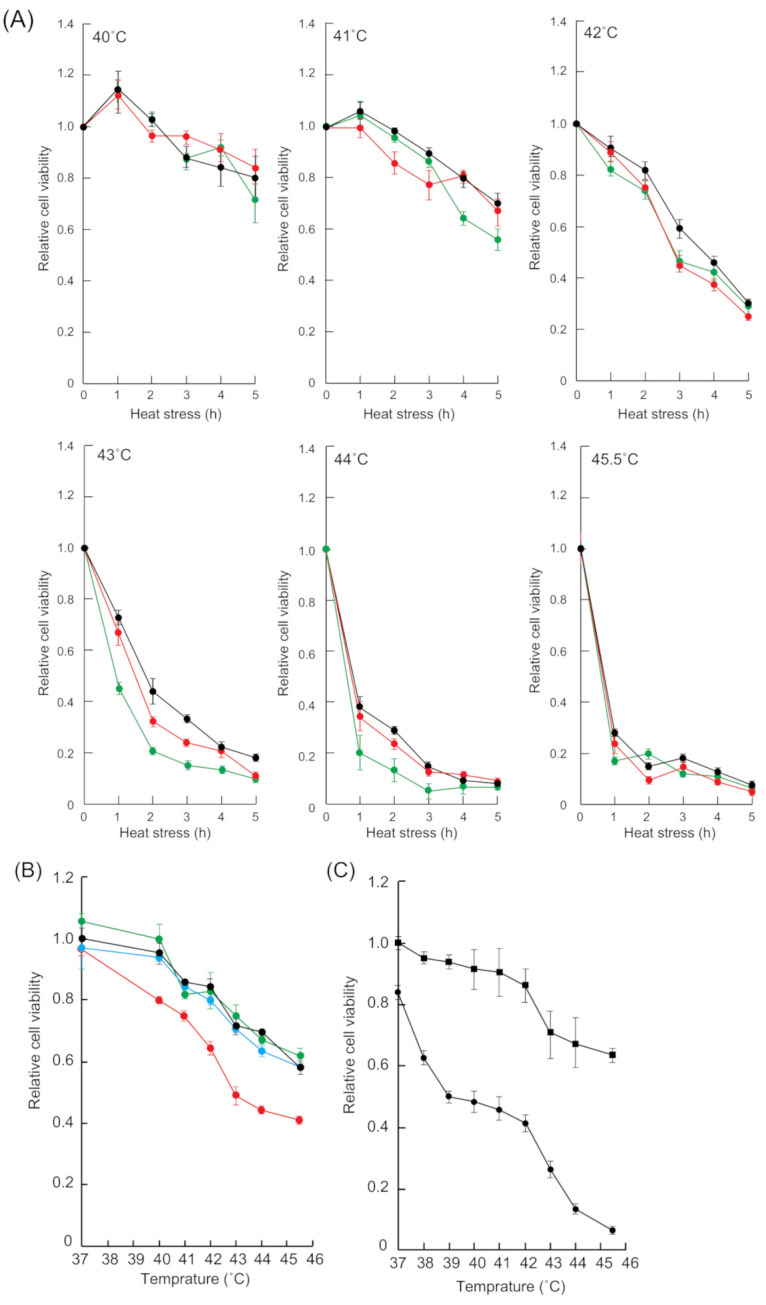
Temperature-dependent cell viability in cells treated with siRNA and compounds. (**A**) The sicontrol- (black), siHSF1- (red), and siSAFB- (green) treated cells were incubated at 40–45.5 °C for 0–5 h. Data represent the means ± SEM of four independent experiments. (**B**) The cells were incubated at 37–45.5 °C for 30 min in the presence of DMSO (black), capsaicin (light blue), SB366791 (red), or GSK2193874 (green). Data represent the means ± SEM of four independent experiments. (**C**) The cells were incubated at 37–45.5 °C for 30 min in the presence of 0.1% 2,5-HD (circle) or in the absence of 2,5-HD (square). Data represent the means ± SEM of six independent experiments.

The cell growth inhibition induced by heat stress depends on heat-stressed time and temperature [40,41,42]. Therefore, we examined whether heat stress-induced cell growth inhibition was enhanced in a time- and temperature-dependent manner in HSF1 and SAFB knockdown cells (Figure 4A). The cell viability patterns of siHSF1- and siSAFB-treated cells at 40 °C and 41 °C were similar to that of sicontrol-treated cells. By contrast, the viability of siHSF1- and siSAFB treated cells at 42 °C was slightly less than that of sicontrol-treated cells. Furthermore, the viability of siHSF- and siSAFB-treated cells at 43 °C and 44 °C was considerably less than that of sicontrol-treated cells.

We then examined whether SB366791 and 2,5-HD enhanced temperature-dependent cell growth inhibition. The cell viability pattern of DMSO-treated cells was similar to that of capsaicin- and GSK2193874-treated cells (Figure 4B). By contrast, the viability of SB366791-treated cells was markedly less than that of DMSO-treated cells. In addition, cell viability in the presence of 0.1% 2,5-HD was markedly lower than that in the absence of 2,5-HD (Figure 4C). These results indicated that the inhibition of HSF1 and SAFB granule formation enhanced temperature-dependent cell growth inhibition.

### 2.6. Recovery Time-Dependent Upregulation of HSP27 and HSP70 Was Inhibited by 2,5-HD

The present study showed that the formation of HSF1 and SAFB granules correlated with cell growth inhibition (Figure 2 and Figure 4). Previous reports have shown that the expression of HSPs, especially HSP70, was involved in thermal resistance [29,35,43]. The expression of HSP27 and HSP70 increases depending on recovery time after heat stress [36,41]. Therefore, we hypothesized that the upregulation of HSPs during recovery might be inhibited by the inhibition of HSF1 and SAFB granule formation, which may lead to the enhancement of cell growth inhibition induced by heat stress.

**Figure 5:**
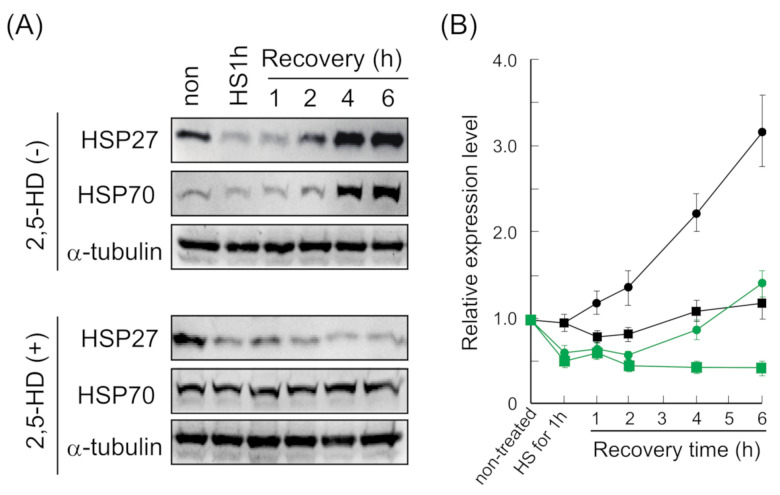
Formation of HSF1 and SAFB granules was involved in the upregulation of HSP27 and HSP70 depending on the recovery time. (**A**) The expression levels of HSP27 and HSP70 were evaluated by using Western blotting during heat stress recovery. α-tubulin was used as a control. (**B**) The ratio of HSP27 and HSP70 was calculated as 1.0 for non-treated time point. HSP27 in the absence of 2,5-HD, green circle; HSP27 in the presence of 2,5-HD, green square; HSP70 in the absence of 2,5-HD, black circle; HSP70 in the presence of 2,5-HD, black square. Data represent the means ± SEM of four independent experiments.

To confirm our hypothesis, the cells were allowed to recover for 1–6 h at 37 °C after 1 h exposure to heat stress in the presence of 0.1% 2,5-HD, and then the expression levels of HSP27 and HSP70 were analyzed (Figure 5A,B). The expression levels of HSP27 and HSP70 in 2,5-HD untreated-cells gradually increased depending on the recovery time, while those of the cells treated with 0.1% 2,5-HD barely increased. These results suggested that the formation of HSF1 and SAFB granules was likely to be involved in the upregulation of HSP27 and HSP70 during heat stress recovery, causing enhanced cell growth inhibition.

### 2.7. Inhibition of HSF1 and SAFB Granule Formation Enhanced Apoptosis Induced by Heat Stress

Apoptosis is induced in cells exposed to heat stress [41,44], while necrosis is induced by exposure to temperatures higher than those required for induction of apoptosis [41]. We examined whether apoptosis induced by heat stress was enhanced in siSAFB-treated cells by staining them with Nucview488 and propidium iodide (PI). Nucview488 detects the activity of caspase3/7, which initiates apoptosis, and therefore, cells stained by Nucview488 and not PI indicate early apoptosis [45], whereas both Nucview488 and PI-stained cells indicate late apoptosis. We detected a few cells stained with PI or Nucview488 in sicontrol- or siSAFB-treated cells incubated at 37 °C, suggesting that lipofectamin reagent might have slightly induced necrosis and apoptosis (Figure S5A,B). By contrast, apoptotic siSAFB-treated cells incubated at 43 °C were markedly increased compared to those in the presence of DMSO.

**Figure 6:**
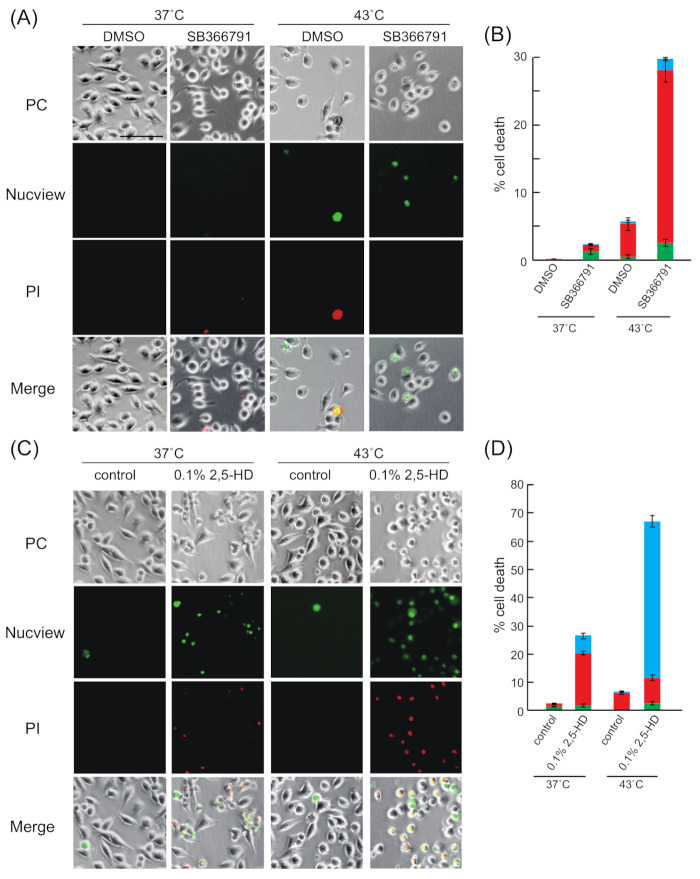
Inhibition of HSF1 and SAFB granule formation enhanced apoptosis induced by heat stress. (**A**) Apoptosis of the cells, which were treated with DMSO or SB366791, was detected using Nucview488 (green) and propidium iodide (PI) (red). Non-apoptotic cell death was detected using PI. (**B**) The ratios of apoptotic cells and non-apoptotic dead cells are shown. Green bars, PI-stained cells; red bars, Nucview-stained cells; blue bars, Nucview- and PI-stained cells. Data represent the means ± SEM of three independent experiments. More than 250 cells were counted in each experiment. Scale bars = 100 µm. (**C**) Apoptosis of the cells treated with 0.1% 2,5-HD was detected using Nucview488 (green) and PI (red). Non-apoptotic cell death was detected using PI. (**D**) The ratios of apoptotic cells and non-apoptotic dead cells are shown. Green bars, PI-stained cells; red bars, Nucview-stained cells; blue bars, Nucview- and PI-stained cells. Data represent the means ± SEM of four independent experiments. More than 1000 cells were counted in each experiment. Scale bars = 100 µm.

We next examined whether apoptosis was enhanced by the combination of heat stress and SB366791 treatment by staining with Nucview488 and PI. We detected few cells stained with only Nucview488, as well as those stained with both Nucview488 and PI at 43 °C in the presence of DMSO, indicating that apoptosis was slightly induced under heat stress (Figure 6A,B). Similarly, a few cells stained with PI or Nucview488 were detected in the presence of SB366791 at 37 °C, indicating that 15 µM SB366791 slightly induced necrosis and apoptosis (Figure 6A,B). By contrast, apoptotic cells in the presence of SB366791 at 43 °C were markedly increased compared to those in the presence of DMSO.

We examined whether apoptosis was enhanced by the combination of heat stress and 0.1% 2,5-HD. The number of apoptotic cells was higher in the presence of 0.1% 2,5-HD at 43 °C compared to that in its absence (Figure 6C,D). This indicated that the combination of heat stress and 2,5-HD induced apoptosis compared to heat stress alone. Taken together, these results suggested that the inhibition of HSF1 and SAFB granule formation was likely to enhance apoptosis induced by heat stress. Moreover, the number of apoptotic cells in the presence of 0.1% 2,5-HD at 37 °C was higher compared to that in its absence. This indicated that the viability of cells treated with 0.1% 2,5-HD was slightly inhibited even at 37 °C as 2,5-HD induced apoptosis (Figure 3A and Figure 6C,D).

As shown in Figure 6C and 6D, the ratio of the cells stained with Nucview488 and PI after 2,5-HD and heat stress treatment was higher than that after treatment with 2,5-HD at 37 °C. These results suggested that the combination of heat stress and 2,5-HD accelerated the progression of apoptosis compared to heat stress alone.

Nucview488 detects only the final caspase 3/7 activity. Therefore, apoptosis in the presence of 2,5-HD was detected using JC-1 dye reagent, which detects depolarization of the mitochondrial membrane. As shown in Figure S6, the JC-1 fluorescence ratio in the presence of 0.1% 2,5-HD at 43 °C was decreased compared to that in the absence of 2,5-HD at 37 °C. This result suggested that the combination of 2,5-HD and heat stress induced apoptosis via the mitochondrial apoptotic pathway. In addition, the JC-1 fluorescence ratio in the presence of 0.1% 2,5-HD at 43 °C was decreased compared to that in the absence of 2,5-HD at 43 °C, suggesting that apoptosis in the presence of 0.1% 2,5-HD at 43 °C was enhanced via the mitochondrial apoptotic pathway.

Finally, we examined whether apoptosis and necrosis were induced in the presence of 2,5-HD during heat stress. As shown in Figure S7, PI-stained cells were not observed among the cells exposed to 2,5-HD for 1–3 h at 37 °C, but were slightly observed among the cells exposed for 4–5 h. In contrast, PI-stained cells in the presence of 2,5-HD during heat stress were gradually increased in a time-dependent manner. In addition, apoptosis in the presence of 2,5-HD during heat stress was not observed, suggesting that 2,5-HD induces necrosis during heat stress. As shown in Figure 6D and Figure S7, apoptosis was mainly detected in the cells that recovered after treatment with 2,5-HD at 43 °C for 1 h, whereas necrosis without apoptosis was slightly detected during heat stress. These results suggested that apoptosis of cells treated with 2,5-HD and heat stress might be mainly induced during heat stress recovery and that necrosis could be mainly induced during heat stress.

## 3. Discussion

**Figure 7:**
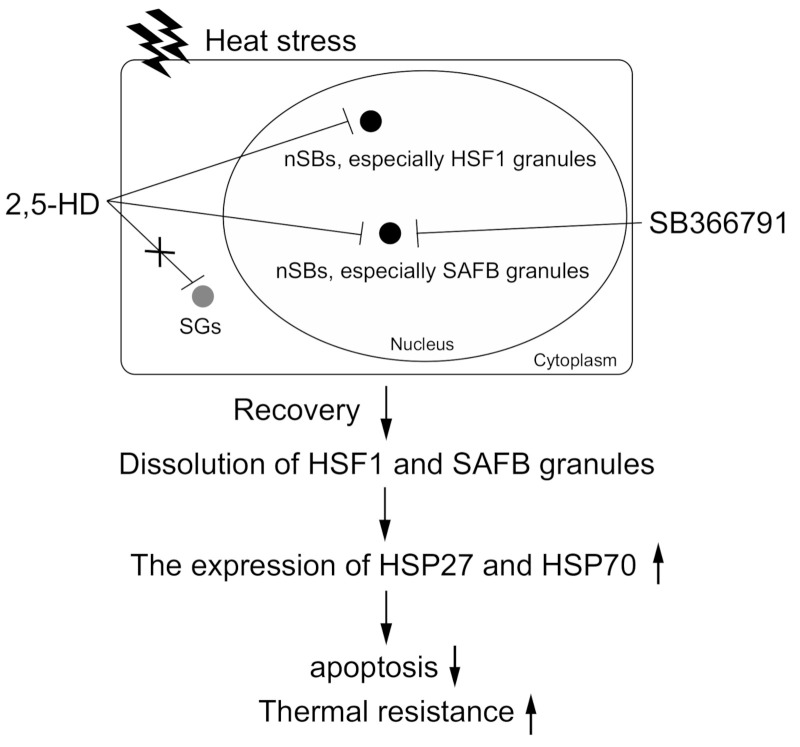
Predicted thermal resistance mechanism via HSF1 and SAFB granule formation. HSF1 and SAFB granules form in the nucleus of cells due to heat stress. The expressions of HSP27 and HSP70 were upregulated (up-arrow) during recovery, resulting that apoptosis was inhibited (down-arrow); that is, thermal resistance was enhanced (up-arrow). In the presence of SB366791 or 2,5-HD, HSF1 and SAFB granule formation was inhibited during heat stress. In addition, the upregulation of HSP27 and HSP70 expression during recovery were barely induced by 2,5-HD, which inhibited HSF1 and SAFB granule formation. As a result, apoptosis was enhanced; that is, thermal resistance was decreased. Therefore, cells exhibit anti-apoptotic function because of the upregulation of HSP27 and HSP70 during recovery; that is, the formation of HSF1 and SAFB granules regulates thermal resistance.

Herein, we found that the knockdown of HSF1 and SAFB enhanced time- and temperature-dependent cell growth inhibition. SB366791, which inhibited the formation of SAFB, and 2,5-HD, which inhibited the formation of HSF1 and SAFB granules, also enhanced the temperature-dependent cell growth inhibition induced by heat stress. Moreover, SB366791 and 2,5-HD enhanced apoptosis induced by heat stress. In addition, the upregulation of HSP27 and HSP70 during heat stress recovery was suppressed by 2,5-hexanediol. Our results suggested that the formation of HSF1 and SAFB granules was likely to be involved in the upregulation of HSP27 and HSP70 during heat stress recovery. Thus, the formation of HSF1 and SAFB granules was involved in thermal resistance (Figure 7).

While HSF1 granules induced by MG132 were reportedly inhibited by 10% 1,6-HD [28], in the present study heat stress-induced HSF1 granule formation was inhibited in the presence of 0.1% 1,6-HD. We envisage two hypotheses that may explain this discrepancy in HSF1 susceptibility: First, it may be attributable to a difference in the intracellular uptake of 1,6-HD under heat stress. Heat stress elevates membrane permeability, increasing the intracellular uptake of chemical compounds [46]. This may cause higher intracellular uptake of 1,6-HD under heat stress, and therefore, the formation of HSF1 granules in the present study was inhibited at a lower concentration of 1,6-HD.

Second, it may be due to a difference in the number of post-translational modification sites, especially phosphorylation sites, of HSF1, which may differ based on induction by heat stress or MG132. Post-translational modification of proteins is important for granule formation via LLPS [47]. While heat stress and MG132 induce post-translational modification of HSF1, the number of HSF1 phosphorylation sites differs in each case. According to the PhosphoSitePlus database (https://www.phosphosite.org/homeAction, 10 May 2020), HSF1 is phosphorylated at six sites under heat stress, but only at two sites in the presence of MG132. Moreover, LLPS of Tau was dependent on hydrophobic interactions [48]. As the hydrophobicity of a protein depends on the number of phosphorylation sites [47], HSF1 granules induced by heat stress might possess weaker hydrophobic interactions compared to those in granules induced by MG132. This may in turn result in inhibition of HSF1 granule formation at lower 1,6-HD concentrations.

The dissolution of HSF1 granules in cells treated with mitoxantrone increased HSF1 activity, which is involved in HSP expression [28]. HSF1 and SAFB granules reportedly disappeared after recovery from heat stress [19,49]. The expression of HSP27 and HSP70 increased in a time-dependent manner during recovery after heat stress [36,41]. Furthermore, the present study showed that while 2,5-HD, which inhibited HSF1 and SAFB granule formation, did not affect the expression of HSP27 and HSP70 during heat stress, it inhibited their upregulation during heat stress recovery. Taken together, the previous reports and our findings suggested that the expression of HSP27 and HSP70 were unaffected during the formation of HSF1 and SAFB granules, and dissolution of these granules was likely to trigger the upregulation of HSP27 and HSP70 during recovery from heat stress.

In the present study, HSP27 and HSP70, which are molecular chaperones, were analyzed. Previous reports have shown that molecular chaperones such as HSP60 and HSP90 are involved in thermal resistance [50,51]. The expression of HSPs, including HSP60 and HSP90, is increased by heat stress. Therefore, further analyses of other HSPs will be needed to completely understand their roles under heat stress in the presence of 2,5-HD.

Hyperthermia therapy is a cancer treatment based on cell growth inhibition and apoptosis induced by heat stress [52,53]. The main mechanism of hyperthermia involves killing of cancer cells via destruction of protein structures by increasing the intracellular temperature. Hyperthermia-induced apoptosis is gradually induced at 39–42 °C and reaches maximum efficiency at 43 °C or more [52,53]. This was also corroborated in the present study where the cell growth inhibition was efficiently induced at 43 °C or more. However, hyperthermia therapy has many inadequacies. For instance, thermal resistance, which involves the expression of HSPs, reduces its therapeutic effect [54]. Additionally, it is difficult to reach and maintain the desired temperature around the tumor in vivo [55]. To overcome these problems, a combination of hyperthermia therapy and sensitizers, which inhibit thermal resistance, was developed [56]. In the present study, 2,5-HD enhanced the temperature-dependent cell growth inhibition induced by heat stress. However, 2,5-HD induced cytotoxicity even under the normal temperature conditions, while SB366791 showed minimum cytotoxicity under normal conditions. Therefore, SB366791 may be a candidate sensitizer with applications in hyperthermia therapy at lower temperatures.

HSF1 granules induced by heat stress are observed in different cell lines such as epithelial, HOS, and A431 cells [17]. In addition, SAFB granules are observed in HEK293 cells [57]. Therefore, the suppression of HSF1 and SAFB granule formation by SB366791 and 2,5-HD might enhance the cell growth inhibition induced by heat stress in these cell lines as well.

The report by Ulianov et al. showed that 5% 1,6-HD, which is structurally similar to 2,5-HD, partially compromises 3D genome organization [58]. Further, 5% and 10% 1,6-HD cause chromatin hyper-condensation, whereas 2.5% 1,6-HD causes nucleosome clustering slightly similar to that in untreated cells [59]. Moreover, frozen chromatin, which is induced by 2.5% 1,6-HD, is restored to frozen by removing 1,6-HD, whereas 5% and 10% 1,6-HD freeze chromatin [59]. In the present study, the concentration of 2,5-HD (0.1 µM) was lower than that used in the reports by Ulianov et al. and Itoh et al. In addition, 2,5-HD was removed from the medium after heat stress; that is, 2,5-HD was not present during heat stress recovery. Therefore, short-term exposure to 0.1% 2,5-HD might not affect chromatin. However, long exposure (over 3 h) to 0.1% 2,5-HD at 37 °C decreased cell viability, suggesting that 2,5-HD with long exposure might affect chromatin. Therefore, further studies, such as those on the effect of 2,5-HD on chromatin structure including the effect on histone modifications, are warranted.

In conclusion, our results suggested that the formation of HSF1 and SAFB granules was involved in apoptosis induced by heat stress. Thus, our study findings provide insights into the mechanism of stress resistance. However, the nSB formation mechanism, including that of HSF1 and SAFB granules, and the upregulation mechanism of HSP27 and HSP70, in which the formation of HSF1 and SAFB was involved, remain unclear. To develop a more effective hyperthermia therapy, it will be necessary to elucidate the detailed mechanisms of nSB formation and HSP27 and HSP70 upregulation.

## 4. Materials and Methods

### 4.1. Cell Culture

HeLa cells were obtained from RIKEN BRC, which is participating in the National Bio-Resource Project of MEXT, Japan. HeLa cells were cultured as described previously [6]. Fetal bovine serum (FBS) was purchased from Sigma (St. Louis, MO, USA).

### 4.2. Treatment with siRNA

For HSF1, HSF2, SAFB, and SAM68 knockdown, siGENOME SMART pools for each molecule were used. For SF2/ASF knockdown, siRNA described by Somberg et al. (2010) was used [60]. All siRNAs were purchased from Dharmacon (Lafayette, CO, USA). HeLa cells were transfected with 10 nM siRNA by using Lipofectamin RNAimax (Invitrogen, Carlsbad, CA, USA) for 24 h at 37 °C. On the next day, the cells were replated onto 48-well plates, and then cultured for a further 24 h.

### 4.3. Immunocytochemistry

HeLa cells were incubated in a water bath at 43 °C for 1 h in the presence of 0.01%–0.1% 1,6-HD (Tokyo Chemical Industry, Tokyo, Japan) or 2,5-HD (Tokyo Chemical Industry, Tokyo, Japan). Immunocytochemistry was performed as described previously [61]. Rabbit anti-HSF1 (dilution 1:1000) was purchased from Cell Signaling Technology (Danvers, MA, USA). Mouse anti-SAF-B (1:200) was purchased from abnova (Taipei, Taiwan). Rabbit anti-eIF3γ (dilution 1:100) was purchased from Santa Cruz Biotechnology (Dallas, TX, USA). Alexa 594- and Alexa 647-conjugated secondary antibodies were purchased from Invitrogen, and 4′,6-diamidino-2-phenylindole (DAPI) was obtained from Dojindo (Kumamoto, Japan). The stained cells were examined using a confocal microscope (FV-1000; Olympus, Tokyo, Japan). The granules of HSF1 and SAFB with an area of more than 0.5 µm^2^ and with high fluorescence intensity were counted. The cells were pretreated with 15 µM SB366791 (Fujifilm Wako, Osaka, Japan) or DMSO for 1 h at 37 °C and then incubated at 43 °C for 1 h in their presence. Immunocytochemistry was performed as described previously herein.

### 4.4. Quantitative PCR (qPCR)

Total RNA was extracted and reverse-transcription was performed, as described previously [6]. qPCR was performed using KOD SYBR qPCR mix (TOYOBO, Osaka, Japan) on a detection system (Applied Biosystems, Foster City, CA, USA) using specific oligo primers listed in Supplemental Table S1. GAPDH was used as an internal control. Relative mRNA expression levels of HSF1, HSF2, SAFB, SF2/ASF, and SAM68 were normalized against the GAPDH expression level by using 2^−ΔΔCt^ method.

### 4.5. Western Blot Analysis

After transfection with specific siRNAs for 24 h, HeLa cells were provided with fresh medium containing 10% FBS and then cultured for 24 h. Total protein was extracted and Western blot analysis was performed, as described previously [62]. Rabbit anti-HSF1 (dilution 1:1000, Cell Signaling Technology, Danvers, MA, USA), rabbit anti-α-tubulin (dilution 1:1000, Cell Signaling Technology, Danvers, MA, USA), mouse anti-HET/SAF-B (dilution 1:2000, Upstate Biotechnology, Frankfurter, Germany), rabbit HSF2 (dilution 1:500, Genetex, Alton Pkwy Irvine, CA, USA), and rabbit SAM68 (dilution 1:1000, abcam, Cambridge, UK) were used. Band intensities were quantified using the image studio (LI-COR, Lincoln, NE, USA).

The cells were incubated using water bath at 43 °C for 1–2 h in the presence of 0.1% 2,5-HD. Western blot analysis was performed as described above. Mouse anti-HSP70 (dilution 1:1000) was purchased from Enzo Life Science (New York, NY, USA). Mouse HSP-27 (dilution 1:1000) was purchased from Cell Signaling Technology (Danvers, MA, USA). Rabbit anti-HSF1 (phospho S326) (dilution 1:5000) was purchased from abcam (Cambridge, UK).

To analyze the expression levels of HSP27 and HSP70 during recovery, the cells were incubated at 43 °C for 1 h in the presence of 0.1% 2,5-HD. After exposure to heat stress, the medium was replaced with fresh medium and then the cells were cultured at 37 °C for 1–6 h. Western blot analysis was performed as described above.

### 4.6. Cell Viability Assay

The cells transfected with siRNA were incubated in a water bath set to the measurement temperature (40–45.5 °C) for 0–5 h. After exposure to heat stress, the cell medium was replaced with fresh medium and the cells were maintained at 37 °C for 24 h in an atmosphere of 5% CO2. Cell viability was measured using cell counting kit-8 (Dojindo, Kumamoto, Japan) in accordance with the manufacturer’s instructions.

To determine inhibition of heat stress, the cells were incubated for 0–5 h in the presence of 0.1% 2,5-HD, and then the cells were maintained at 37 °C for 24 h. Cell viability was measured using the cell counting kit-8.

The cells were pretreated with 15 µM Capsaicin (Fujifilm Wako, Osaka, Japan), 15 µM SB366791, or 300 nM GSK2193874 (Cayman Chemical, Ann Arbor, MI, USA) for 1 h at 37 °C, and then incubated at the measurement temperature for 0–5 h in their presence. After exposure to heat stress, the cells were maintained at 37 °C for 24 h in an atmosphere of 5% CO2. Cell viability was measured using cell counting kit-8.

siHSF1- and siSAFB-treated cells were incubated in the presence of 0.1% 2,5-HD at 43 °C for 30 min. After exposure to heat stress, the cells were maintained at 37 °C for 24 h. Cell viability was measured using cell counting kit-8.

### 4.7. Detection of Apoptotic and Necrotic Cells

The cells were pretreated with DMSO or 15 µM SB366791 for 1 h at 37 °C, and then incubated at 37 °C or 43 °C for 1 h in the presence of DMSO or SB366791, respectively. After exposure to heat stress, the cells were maintained at 37 °C for 24 h. To detect apoptotic and necrotic cells, Nucview488 (Biotium, Fremont, CA, USA) and PI (Molecular probes, Eugene, OR, USA) were used according to the manufacturer’s instructions. Apoptotic or necrotic cells were examined using a fluorescence microscope (IX-51; Olympus, Tokyo, Japan).

The cells were incubated at 37 °C or 43 °C for 1 h in the presence of 0.1% 2,5-HD, and then maintained at 37 °C for 24 h. Apoptotic and necrotic cells were detected using Nucview488 and PI.

siSAFB-treated cells were incubated at 43 °C for 1 h and then maintained at 37 °C for 24 h. Apoptotic and necrotic cells were detected using Nucview488 and PI.

The cells were incubated at 37 °C or 43 °C for 0–5 h in the presence or absence of 0.1% 2,5-HD. After heat stress, apoptotic and necrotic cells were detected using Nucview488 and PI.

### 4.8. Detection of Polarization of Mitochondrial Membrane

The cells were incubated at 37 °C or 43 °C for 1 h in the presence of 0.1% 2,5-HD and then maintained at 37 °C for 24 h. To observe the polarization of the mitochondrial membrane, the JC-1 mitochondrial membrane potential assay kit (Cayman Chemical, Ann Arbor, MI, USA) was used according to the manufacturer’s instructions. The fluorescence images of JC-1 aggregates [red] and monomers [green] were examined using a fluorescence microscope (IX-51). The fluorescence intensities were calculated using CellSens software version 1.6 (Olympus, Tokyo, Japan).

### 4.9. Statistical Analyses

The number of HSF1 and SAFB granules was quantified using Fluoview software (Olympus, Tokyo, Japan). The number of apoptotic and necrotic cells was also quantified using Fluoview software version 4.2. All results were expressed as means ± SEM. R software [63] was used for the statistical analyses. The number of experimental replications is shown in the respective figure legends.

## Data Availability

Please refer to suggested Data Availability Statements in section “MDPI Research Data Policies” at https://www.mdpi.com/ethics.

## Supplementary Materials

Supplementary materials can be found at https://www.mdpi.com/article/10.3390/ijms22094982/s1.
