# Supplementary material for: Inhibition of HSF1 and SAFB Granule Formation Enhances Apoptosis Induced by Heat Stress
Source: Int J Mol Sci. 2021 May 7;22(9):4982. doi: 10.3390/ijms22094982 (PMC8124827; doi:10.3390/ijms22094982)
Supplement: Supplementary file 1 [file ijms-22-04982-s001.zip › ijms-1163895-SI.pdf]

Table S1. Primer sequences used for quantitative PCR

Target gene	Primer sequence
HSF1	Fw: 5'-CAAGCTGTGGACCCTCGT
	Re: 5'-TCGAACACGTGGAAGCTGT
HSF2	Fw: 5'-GGAGGAAACCCACACTAACG
	Re: 5'-ATCGTTGCTCATCCAAGACC
SAFB	Fw: 5'-CGGCAACAAGAGCGTTTT
	Re: 5'-TCGTCAGGATTACCACCTTCA
SF2/ASF	Fw: 5'-GCGGTCTGAAAACAGAGTGG
	Re: 5'-TTTAAATCCTGCCAACTTCCA
SAM68	Fw: 5'-CCTGTCAAGCAGTATCCCAAG
	Re: 5'-TTGTATTCCCTTGTGGTCCAA
GAPDH	Fw: 5'-ATTCCACCCATGGCAAATTC-3'
	Re: 5'-GGGATTTCCATTGATGACAAGC-3'

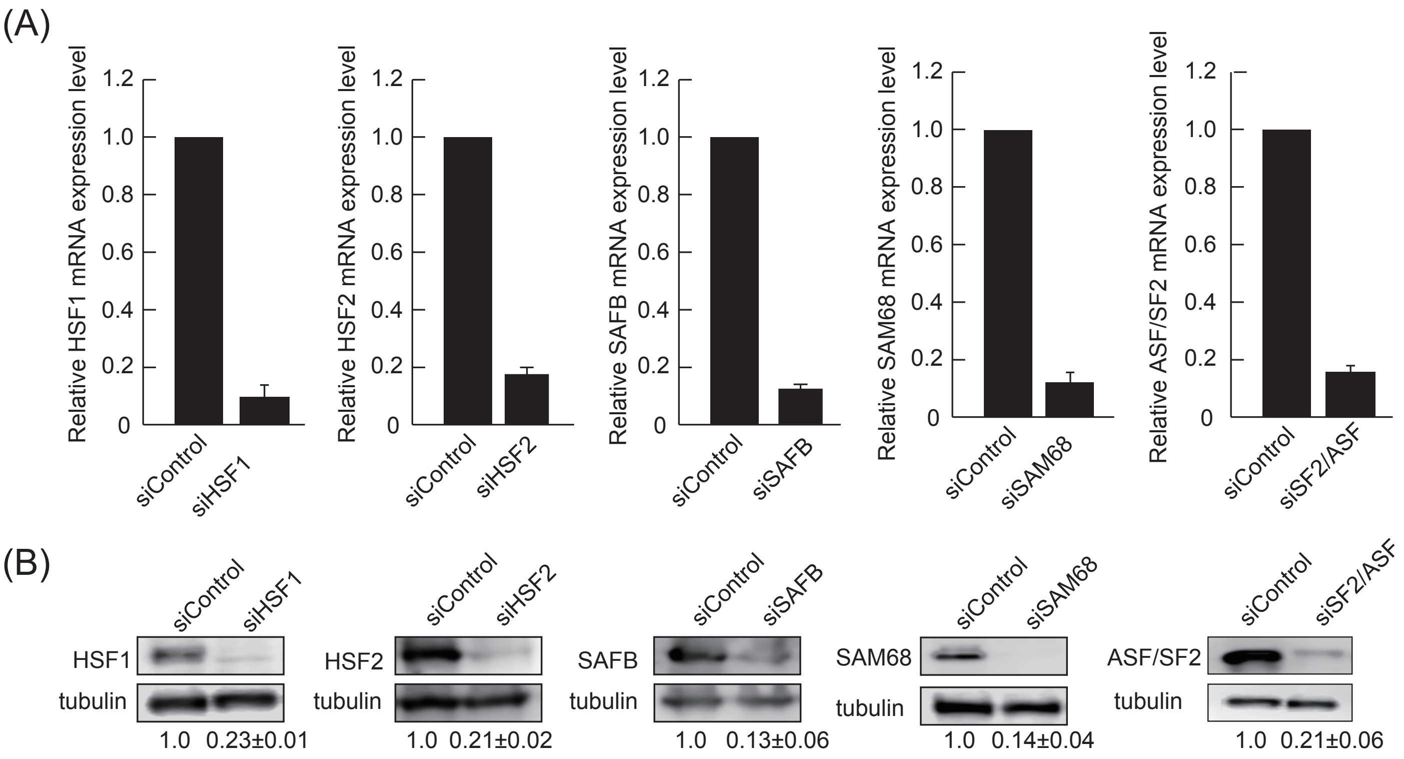

Figure S1. Knockdown efficiencies of HSF1, HSF2, SAFB, SAM68, and SF2/ASF.
(A) mRNA expression levels of HSF1, HSF2, SAFB, SAM68 and SF2/ASF by knockdown were analyzed by qPCR. HeLa cells were transfected with non-targeting siRNA (sicontrol), siHSF1, siHSF2, siSAFB, siSAM68 or siSF2/ASF. Data represent the means ± SEM of three independent experiments. (B) Efficiencies of knockdown using specific siRNA was determined by western blot analysis. Data represent the means ± SEM of three independent experiments.

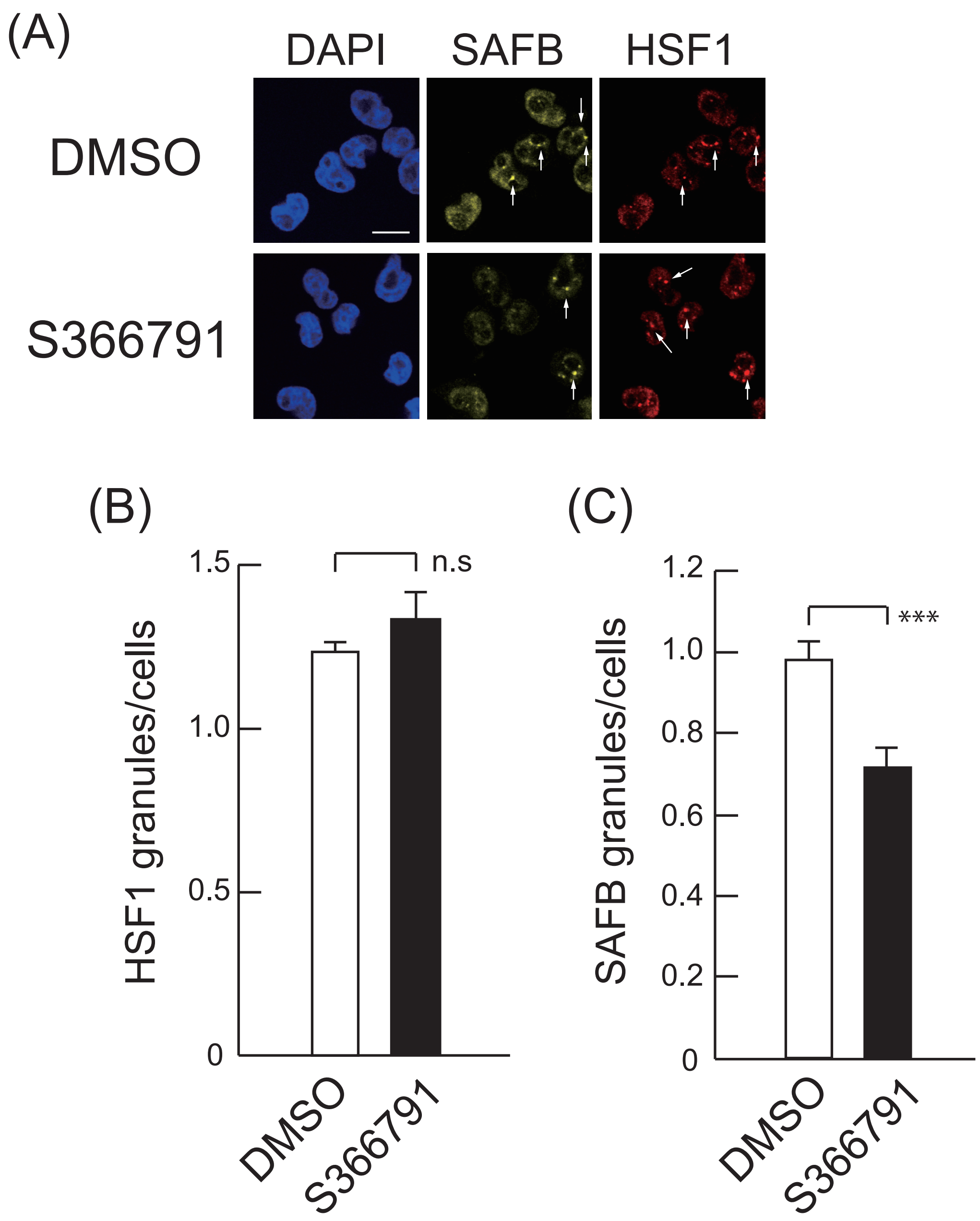

Figure S2. Effect of SB366791 on HSF1 and SAFB granule formation.

HeLa cells were exposed to heat stress at 43 ° C for 1 h in the presence of 15 μM SB366791 or DMSO. HeLa cells were stained for HSF1 (red), SAFB (yellow) and 4′,6-diamidino-2-phenylindole (DAPI) (blue). The arrows indicate HSF1 or SAFB granules. Scale bars = 10 μm.

The number of HSF1 granules (B) or SAFB granules (C) per cell is shown. More than 250 cells were counted in each experiment. Data represent the means ± SEM of six independent experiments. ***$P < 0.001$; P-values were calculated using one-way ANOVA and Dunnett' s test by comparing DMSO with SB366791.

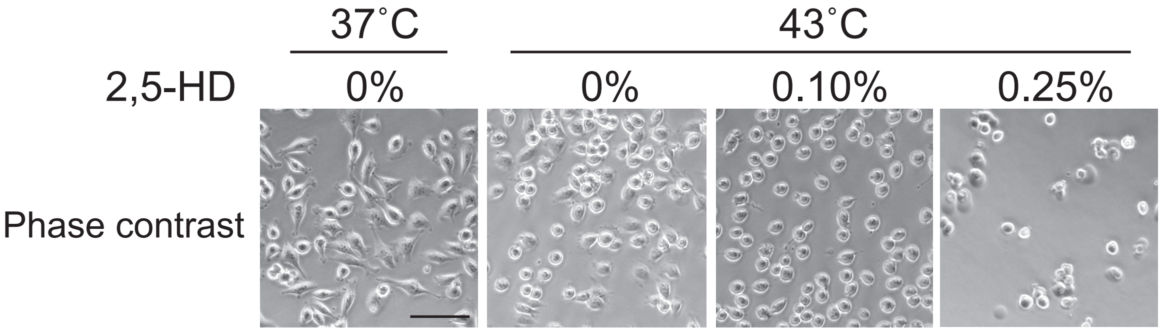

Figure S3. Effect of high concentration 2,5-HD.

The cells were treated with each concentration of 2,5-HD at 37˚C or 43˚C for 1 h. The cells were observed by microscope. Scale bars = 10 μm.

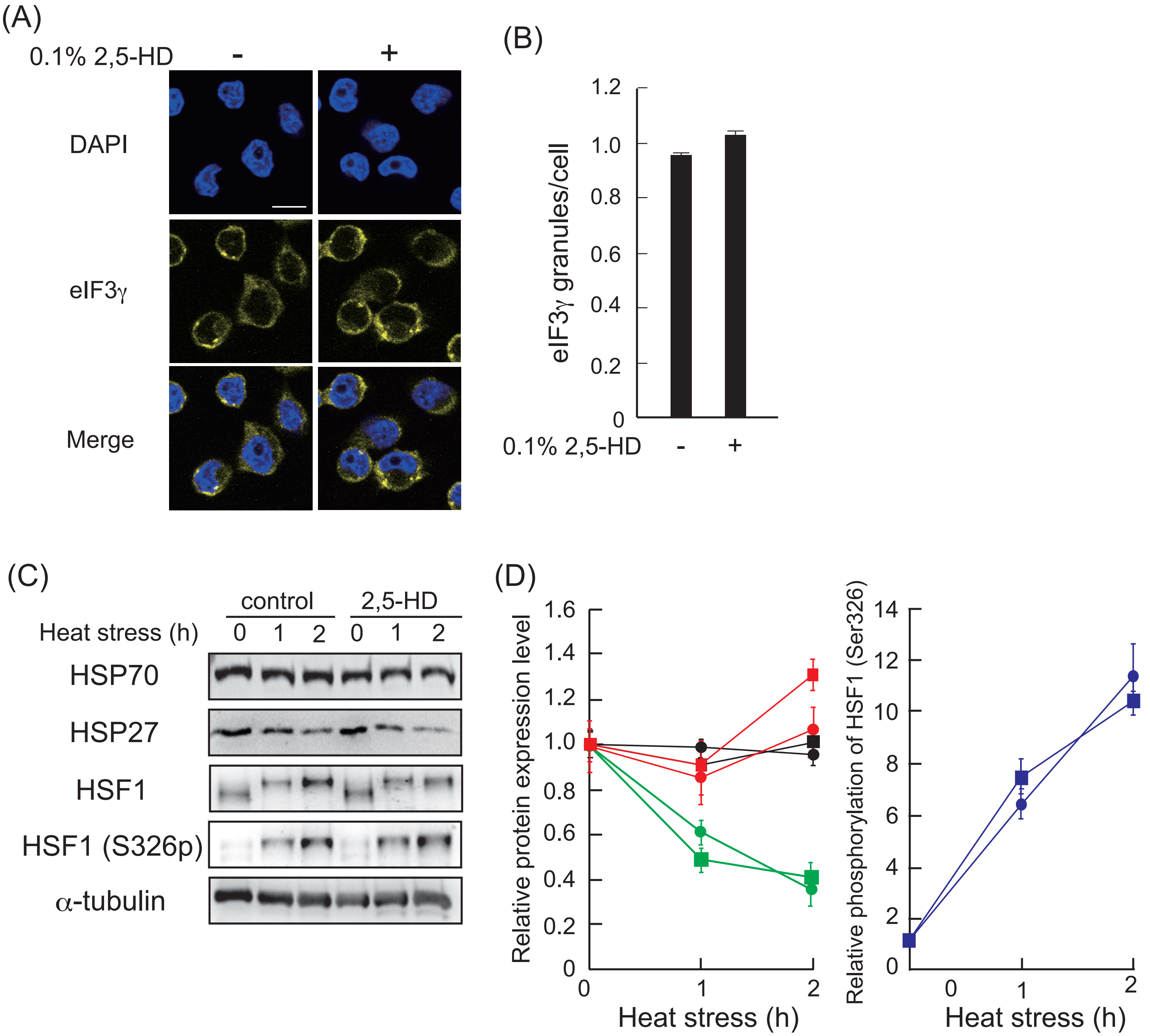

Figure S4. Effect of 2,5-HD for eIF3γ granule formation and the expression of HSP27 and HSP70 and phosphorylated HSF1.

(A) HeLa cells were exposed to heat stress at 43˚C for 1h in the presence of 0.1% 2,5-HD. Images of HeLa cells stained for eIF3γ (yellow) and DAPI (blue). Scale bars = 10 µm. (B) The number of eIF3γ granules per cell was shown. Data represent the means ± SEM of three independent experiments. (C) The expression levels of HSP70, HSF1 and phophorylated HSF1 (S326) were evaluated in the presence of 0.1% 2,5-HD. α-tubulin was used as a control. (D) The ratio of HSP70, HSP27, HSF1 and phophorylated HSF1 (S326) was calculated as 1.0 for non-treated zero time point. HSP70 in the absence of 2,5-HD (control); black circle, HSP70 in the presence of 2,5-HD; black square, HSP27 in the absence of 2,5-HD (control); green circle, HSP27 in the presence of 2,5-HD; green square, HSF1 in the absence of 2,5-HD(control); red circle, HSF1 in the presence of 2,5-HD; red square, phosphorylated HSF1 (S326) in the absence of 2,5-HD(control); blue circle, phosphorylated HSF1 (S326) in the presence of 2,5-HD; blue square. Data represent the means ± SEM of three independent experiments.

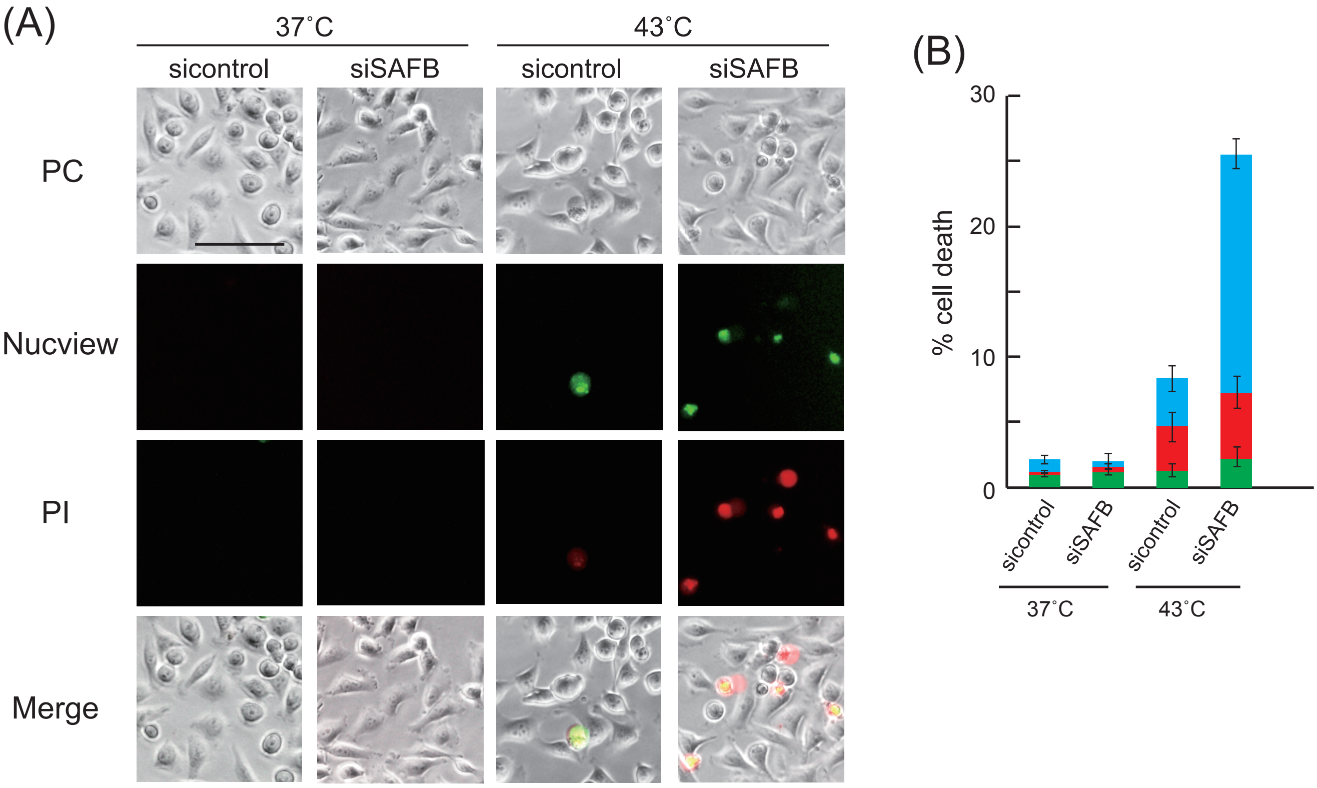

Figure S5. Apoptosis induced by heat stress was enhanced in siSAFB-treated cells.

(A) Apoptosis of the cells, which were treated with sicontrol or siSAFB, was detected using Nucview488 and propidium iodide (PI). Non-apoptotic cell death was detected using PI.

(B) The ratio of apoptotic cells and non-apoptotic dead cells are shown. Green bars, PI-stained cells; red bars, Nucview-stained cells; blue bars, Nucview- and PI-stained cells. Data represent the means ± SEM of three independent experiments. More than 250 cells were counted in each experiment. Scale bars = 100 μm.

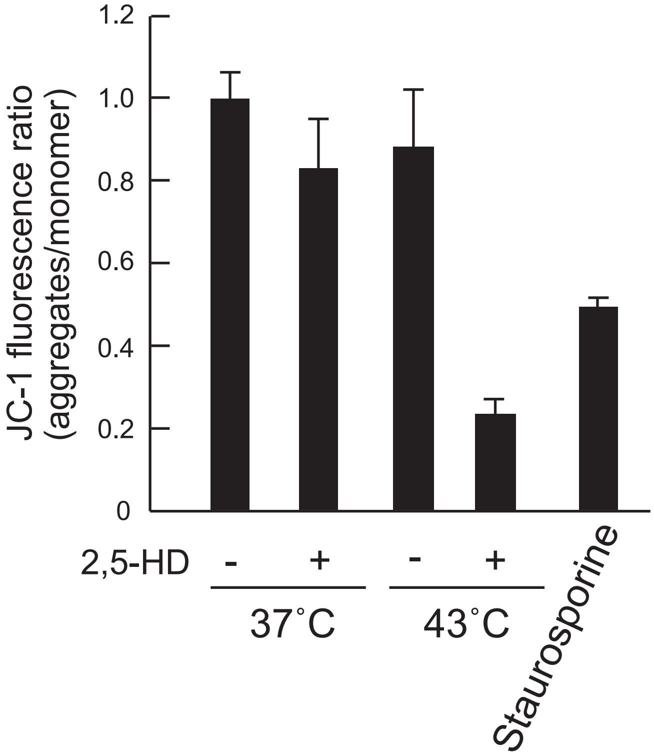

Figure S6. Detection of apoptosis by using JC-1 dye in the presence of 2,5-HD.
HeLa cells were treated with 2,5-HD at 37˚C or 43˚C for 1 h. After treatment, the cell medium was replaced with a fresh medium without 2,5-HD and the cells were maintained at 37˚C for 24 h in an atmosphere of 5% CO_2. Apoptosis was detected usign JC-1 dye. The fluorescence images of JC-1 aggregates [red] and monomers [green] were examined using fluorescence microscope. JC-1 fluorescence ratio of cells, which is maintained at 37˚C in the absence of 2,5-HD, was used as a control for 1.0. Staurosporine was used as a positive control. Data represent the means ± SEM of four independet experiments.

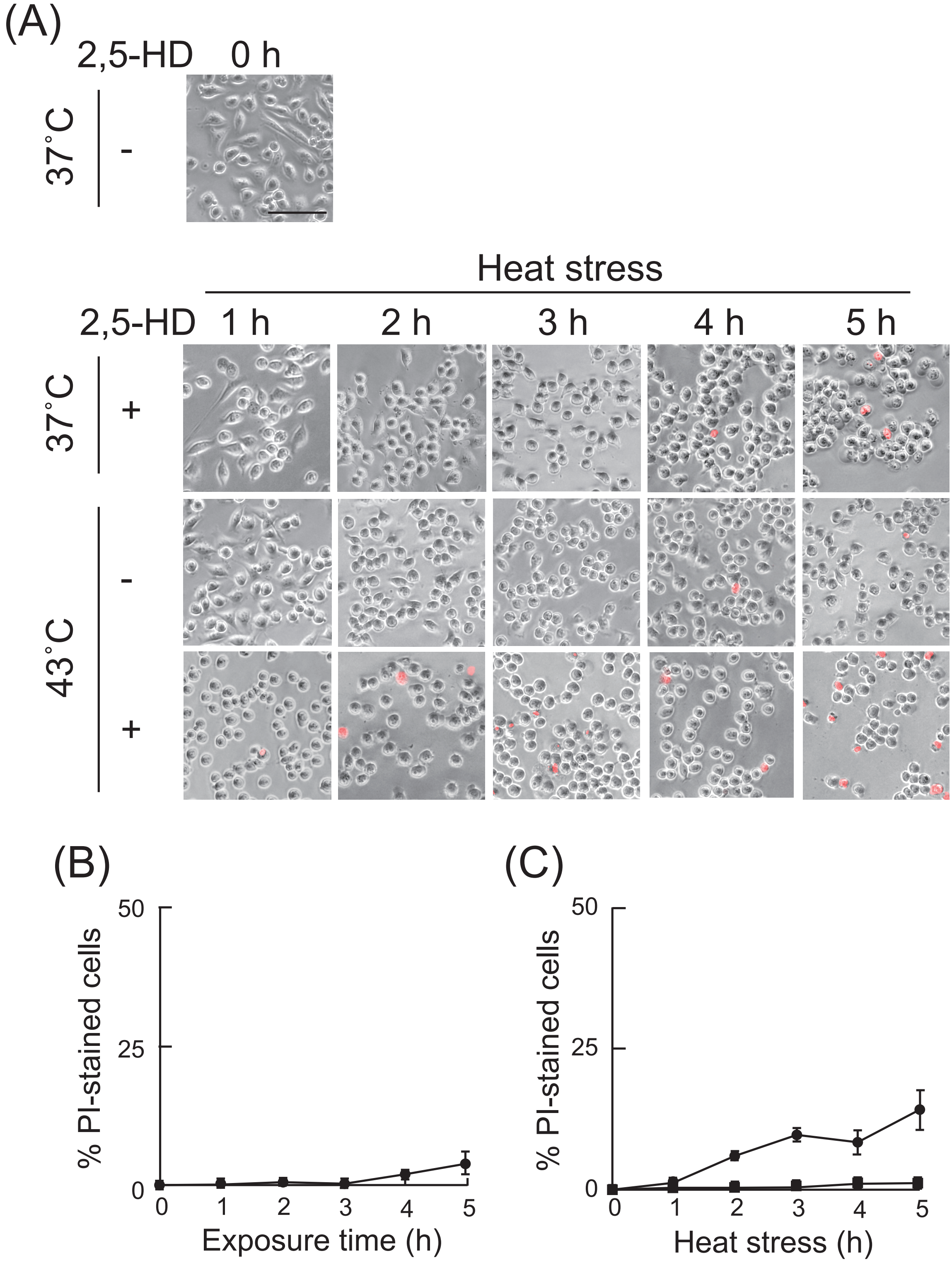

Figure S7. Cell death was slightly induced in the presence of 2,5-HD at 43˚C.

(A) Cell death of the cells, which were treated with 0.1% 2,5-HD at 37˚C or 43˚C for 0-5 h, was detected using Nucview488 (green) and PI (red). Non-apoptotic cell death was stained by PI. Scale bars = 100 μm.

(B) The ratio of non-apoptotic dead cells (PI-stained cells) in the presence of 2,5-HD at 37˚C is shown. Apoptotic cells were not observed. Data represent the means ± SEM of three independent experiments.

(C) The ratio of PI-stained cells in the presence of 2,5-HD (circle) or absence of 2,5-HD (square) at 43˚C is shown. Apoptotic cells were not observed. Data represent the means ± SEM of three independent experiments.
